# Biomimetic Cardiac Tissue Models for In Vitro Arrhythmia Studies

**DOI:** 10.3390/biomimetics8060487

**Published:** 2023-10-14

**Authors:** Aleria Aitova, Andrey Berezhnoy, Valeriya Tsvelaya, Oleg Gusev, Alexey Lyundup, Anton E. Efimov, Igor Agapov, Konstantin Agladze

**Affiliations:** 1 Laboratory of Experimental and Cellular Medicine, Moscow Institute of Physics and Technology, 141700 Dolgoprudny, Russia; 2 M.F. Vladimirsky Moscow Regional Clinical Research Institute, 129110 Moscow, Russia; 3 Almetyevsk State Oil Institute, 423450 Almetyevsk, Russia; 4 Regulatory Genomics Research Center, Institute of Fundamental Medicine and Biology, Kazan Federal University, 420018 Kazan, Russia; 5 Life Improvement by Future Technologies (LIFT) Center, 143025 Moscow, Russia; 6 Intractable Disease Research Center, Graduate School of Medicine, Juntendo University, Tokyo 113-8421, Japan; 7 RUDN University, 117198 Moscow, Russia; 8 Academician V.I. Shumakov National Medical Research Center of Transplantology and Artificial Organs, Ministry of Health of the Russian Federation, 123182 Moscow, Russia

**Keywords:** cardiac tissue, cardiomyocytes, ion currents, excitation wave, ips cells, arrhythmias, reentry, cardiac model

## Abstract

Cardiac arrhythmias are a major cause of cardiovascular mortality worldwide. Many arrhythmias are caused by reentry, a phenomenon where excitation waves circulate in the heart. Optical mapping techniques have revealed the role of reentry in arrhythmia initiation and fibrillation transition, but the underlying biophysical mechanisms are still difficult to investigate in intact hearts. Tissue engineering models of cardiac tissue can mimic the structure and function of native cardiac tissue and enable interactive observation of reentry formation and wave propagation. This review will present various approaches to constructing cardiac tissue models for reentry studies, using the authors’ work as examples. The review will highlight the evolution of tissue engineering designs based on different substrates, cell types, and structural parameters. A new approach using polymer materials and cellular reprogramming to create biomimetic cardiac tissues will be introduced. The review will also show how computational modeling of cardiac tissue can complement experimental data and how such models can be applied in the biomimetics of cardiac tissue.

## 1. Introduction

The cardiovascular system relies on two physiological systems: the myocardial contractility and the electrical excitation conduction through the cardiac tissue. The myocardial contractility enables blood pumping, while the electrical excitation ensures synchronized heart muscle contraction. Most cardiovascular arrhythmias result from disturbances in excitation conduction, leading to uncoordinated muscle fiber contraction. These disturbances are studied in electrophysiological experiments on heart preparations, which are either whole isolated perfused hearts or separate cardiac tissue slices. The main limitation of these studies is that the heart preparations gradually deteriorate, and their long-term survival, which guarantees the stability of physiological processes in the preparations, is unfeasible. In the last two decades, new approaches to studying arrhythmias in cardiac tissue culture models have been developed. These approaches have allowed a deeper understanding of arrhythmia mechanisms and new methods for their treatment.

Tissue models are in vitro multicellular systems that consist of cardiac cells arranged in two- or three-dimensional structures. They can be derived from primary cardiac cells (from animals), pluripotent stem cell-derived cardiac cells, or immortalized cardiac cell lines. Tissue models have several advantages over conventional single-cell models or animal models for studying cardiac arrhythmias [1,2]. They can

- Explore the mechanisms of arrhythmias in detail by isolating and examining individual cells or small cell clusters;
- Evaluate new drugs and treatments for arrhythmias by simulating the conditions in the heart and monitoring how they affect the heart’s electrical activity;
- Investigate the effects of different environmental factors on arrhythmias by exposing cells to various chemicals, toxins, and other environmental factors and measuring how they affect the heart’s electrical activity.

In summary, tissue culture models can recreate the structural and functional heterogeneity of the human heart tissue, as well as allow for precise control and manipulation of the cellular and extracellular environment. They enable high-throughput screening of drugs and gene therapies, and they reduce the ethical and practical issues associated with animal experiments.

Tissue models have been used to investigate various types of cardiac arrhythmias, such as atrial fibrillation, ventricular tachycardia, and catecholaminergic polymorphic ventricular tachycardia (CPVT). For example, researchers have created tissue models of CPVT, a rare inherited arrhythmia that can cause sudden cardiac death in young people [3]. They used induced pluripotent stem cells (iPSCs) from patients with CPVT to generate cardiac cells that exhibited abnormal calcium handling and spontaneous arrhythmias [4]. They then used these cells to create two-dimensional monolayers or three-dimensional engineered heart tissues that replicated the patients’ arrhythmias in vitro. Furthermore, they tested a gene therapy approach that corrected the underlying mutation and restored normal calcium handling and rhythm in a mouse model of CPVT3 [5].

However, while tissue models are promising tools for advancing our knowledge of cardiac arrhythmogenesis and developing novel therapeutic strategies, they also face some challenges and limitations, such as the following:

- The challenge of replicating the exact cellular composition and architecture of the human heart tissue;
- The variability and immaturity of iPSC-derived cardiac cells compared to native cardiac cells;
- The lack of standardized protocols and criteria for producing and assessing tissue models;
- The need for further validation and translation of tissue model findings to human clinical settings.

**Figure 1:**
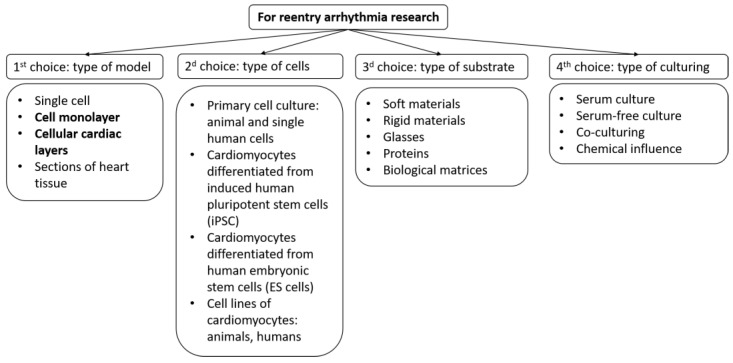
Selection scheme for creating a model of cardiac tissue to study arrhythmias. The figure highlights those models on which this review focuses.

Therefore, future research should aim to improve the quality and reproducibility of tissue models, combine them with other technologies such as tissue engineering, gene editing, optogenetics, and computational modeling, and establish their relevance and applicability for human cardiac arrhythmia research and therapy. To create a biomimetic of cardiac tissue, several parameters of the system could be varied (Figure 1):

- Cell type;
- Type of substrates and coatings for cell seeding;
- Type of growth medium and external factors.

An important role in the creation of artificial tissue is played by the purpose of its creation. In this review, we focus on testing the arrhythmogenicity of the tissue itself or drugs. But even in such cases, for a complete picture of arrhythmogenicity, it is necessary to vary the conditions under which testing is performed. The following are two main variations:

- Simulation of a conduction case in real heart tissue: fibrosis, anisotropy, domain location, and more;
- Congenital mutations leading to arrhythmias.

This short review presents our work in the field of cardiac tissue engineering as an example of creating effective models for the study of cardiac arrhythmias. We developed a novel approach that uses polymeric materials and cellular reprogramming to create cardiac tissues that are structurally and functionally close to native cardiac tissue. In addition, this review will show examples of cardiac tissue modeling and predicting the occurrence of reentry arrhythmias:

- Using different types of cells and rationale for their choice;
- By modeling the structural features of the tissue;
- Using various substrates and coatings;
- Using computer models based on experimental data.

## 2. Reentry Arrhythmia in Cardiac Tissue Models 

**Figure 2:**
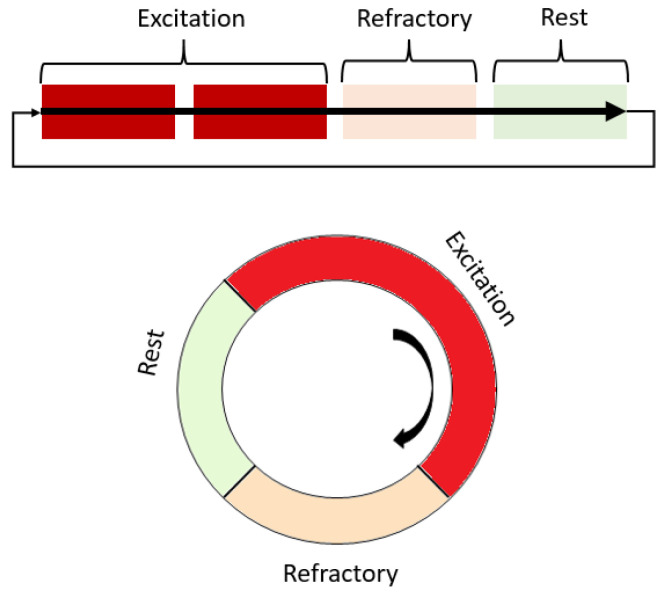
Scheme of the operation of the excitation and spiral wave according to the states that a single element of the excitable medium passes through. Red shades highlight areas that are non-excitable during work or partially excitable.

Cardiac arrhythmia refers to any deviations from the normal electromechanical functioning of the heart, which involves disturbances in the generation and propagation of a cardiac impulse [6]. The main disturbances in impulse propagation result from various conduction blockades and reentrant rhythms, which occur when a cardiac impulse re-excites a certain area of the myocardium under certain conditions. Re-entry causes the most dangerous cardiac arrhythmias, such as ventricular tachycardia and atrial and ventricular fibrillation, which may be associated with spiral waves and may occur after heart attacks or strokes [7,8]. Re-entry can be either ordered when the impulse travels along a fixed anatomical path or random when the path is constantly changing [9]. Fibrillation is a phenomenon of multiple random re-entry circuits in an excitable medium such as cardiac tissue. The conditions for maintaining re-entry in an excitable medium include prolonging the conduction time or shortening the effective refractory period [10]. Spiral waves have also been observed in various autocatalytic types of chemical reactions, such as the Belousov–Zhabotinsky reaction, and biological systems [10,11,12]. When the ratio of speed, refractory period, and obstacle radius is certain, a wave can circulate an element of the medium located at the edge of the obstacle several times [13,14]. Re-entry as a fundamental reason for arrhythmias can also be studied in simulated cardiac tissue models (Figure 2).

Rotating waves, or reentries, are a type of abnormal electrical activity in the heart that can lead to many life-threatening cardiac tachyarrhythmias. Antitachycardia or overdrive pacing (ATP) is a technique that delivers rapid electrical stimuli to the heart to restore normal rhythm [15,16,17]. However, the exact mechanism of how ATP works is not fully understood and remains an active area of research. This is because studying this phenomenon poses two main challenges. First, it is hard to predict how electrical waves propagate in a real heart, especially when it is affected by diseases that make it more complex and heterogeneous. Second, it is technically difficult to record the propagation patterns in a real heart with high enough temporal and spatial resolution to reveal the fine details of how rotating waves and paced wave fronts interact. Therefore, most of our knowledge about the mechanisms of reentry termination by an external source initially comes from numerical studies [18].

Using simplified models of cardiac tissue, such as thin layers of cardiac cells grown in culture, we were able to gain some insights into the mechanism of ATP. These studies have examined how pacing affects reentrant activity and have provided insights into the motion of a spiral tip, which is the point where the rotating wave curls around itself. The motion of the spiral tip is relevant to the idea of paced-induced spiral drift, which is a potential mechanism of ATP that involves moving the spiral away from its original location by pacing. In most cases (about 80%), we successfully terminated functional reentry by overdrive pacing [19,20]. We used confocal microscopy to record images with high spatiotemporal resolution and track the trajectory of a spiral wave tip in an interactive mode [21]. We visualized how a spiral wave tip interacted with the wavefront of paced waves in cardiomyocyte cultures. Our data suggest that (1) stable spiral waves in cardiac monolayers tend to be pinned to local microheterogeneity, which are small areas with different electrical properties than their surroundings; (2) overdrive pacing can shift a rotating wave from its original site by colliding with its tip and causing a wave break; and (3) the wave break, formed as a result of the interaction between the spiral tip and the paced wavefront, can be moved by a pace-induced drift mechanism to an area where it becomes unstable or collides with a boundary and terminates.

The above experiments with cultured cardiac myocytes have suggested that one possible mechanism of spiral wave termination by ATP is the induced drift of the rotating wave and collision with the tissue boundary [22,23]. However, this mechanism may not apply to pathological rotating waves in the heart tissue, which are not “free spirals” but rather “pinned spirals”. Due to the heterogeneity of the heart tissue, spiral waves tend to attach to local variations in tissue excitability and become stabilized as pinned rotating waves [24,25]. Tung and co-workers showed that rotating spirals can spontaneously pin to relatively small obstacles in cardiac myocytes [26]. Therefore, unpinning rotating waves from anatomical obstacles is a key step for the success of ATP.

Excitation waves have a minimum velocity to propagate successfully [27]. Propagation failure may occur when the wavefront velocity near the obstacle drops below this critical value [28]. Two factors can reduce the wavefront velocity: increased front curvature and high-frequency pacing. The first factor is that the wave velocity depends on the front curvature in two-dimensional excitable media [25,29]. When a paced wave attaches to an obstacle, it deforms and slows down because of the curvature effect. Moreover, the front curvature increases closer to the center of a spiral wave. Since the obstacle acts as a core for the pinned spiral wave, the wavefronts near the obstacle are always slower and reach the critical minimum velocity faster than the outer wavefronts. Thus, the wave fails to propagate close to the obstacle under a sufficient pacing rate, which results in a detachment or “unpinning” of the wave. After that, the spiral wave drifts according to the described mechanisms [30]. As for the pacing frequency for unpinning and successful ATP, it must be higher than a critical one. The frequency window for successful ATP of pinned rotating waves is narrower than for the case of free rotating waves, and it is more narrow for larger radii of the obstacle. For unpinning and successful ATP, the pacing frequency must be higher than a critical one. The frequency window for the successful ATP of pinned rotating waves is narrower than for free rotating waves, and it narrows further for larger obstacle radii.

In addition to ATP, the problem of spiral wave unpinning can also be solved by various non-invasive methods, which also make it possible in the future to eliminate reentry as the cause of certain types of arrhythmias. These methods are usually based on controlling cardiac excitability on the sensitivity of cells to light and include optogenetic approaches and photocontrol mediated by photosensitive substances. In particular, synthetic photosensitive molecules are used that are capable of photoisomerization upon absorption of photons. For example, such substances are azobenzene and its derivatives [31]. In our works, we tried to determine how effective the photocontrol method can be for eliminating arrhythmias [32,33]. We investigated the effect of a simulated azobenzene trimethylammonium bromide excitability gradient in cultured heart cells on the behavior and termination of reentry waves. Experimental data indicate a shift of the reentry wave mainly towards lower excitability.

In addition to approaches that simulate various excitability of cardiac tissue using substances, approaches based on genetic changes exist. The following study [34] shows that spiral waves in the monolayers of atrial cardiomyocytes can be effectively terminated using a light-induced depolarizing current generated by the arrhythmogenic substrate itself with optogenetic engineering. This result was shown in experiments using neonatal rat cardiac tissue by vector insertion of light-dependent rhodopsin into the tissue, directly affecting the dynamics of calcium.

Another study proposed a fundamentally new phenomenological concept that involves artificially dragging spiral waves by their cores to prove that manipulating the core of a spiral wave should lead to complete spatiotemporal control over its dynamics. The study was performed with in vitro testing on optogenetically modified monolayers of rat atrial cardiomyocytes [35]. As a result, it was possible to achieve reentry termination in several cases compared to the control, which confirms the concept of shockless defibrillation.

## 3. Modeling of Structural Features of Cardiac Tissue to Study the Occurrence of Reentry

As we have already shown, defects in the heart tissue are one of the reasons for the appearance of spatio-temporal structures that disrupt the normal conduction of the excitation wave and the heart rhythm. Defects lead to different types of reentry, most often through a unidirectional conduction block, which will be discussed in the next section, or due to the detachment of the wavefront from the defect boundary [36,37]. Ventricular tachycardia is caused by such an avulsion when the defect becomes the center of spiral wave anchoring [38]. Defects most often represent a naturally formed formation of non-excitable tissue. In modeling, defects are called inhomogeneities, which are zones of a non-excitable medium that have no connection with the conductive part, which is a kind of conduction boundary.

One of our studies aims to understand how tissue curvature affects the propagation of excitation to advance the field of cardiac tissue engineering and to investigate the arrhythmia mechanisms related to the topology and function of the cell network [36]. To conduct this research, we created microgroove arrays with curved and flat surfaces, cultured cardiac tissue on these surfaces, and measured the conduction velocities in both curved and flat tissue samples. The results show a notable decrease in conduction velocity in curved tissue as compared to flat tissue. Additionally, we observed the changes in action potential morphology and alterations in the expression of ion channel proteins in the curved tissue, indicating underlying mechanisms at play. The observed changes further support the notion that tissue curvature affects the underlying mechanisms of electrical conduction. These findings suggest that curvature-induced changes in cellular and extracellular matrix organization play a crucial role in altering the conduction properties of cardiac tissue. To explain these findings, the authors propose a mechanistic model based on the differential growth of tissue along the curved substrate. This leads to altered cellular and extracellular matrix organization, ultimately impacting electrical conduction. The model is further supported by additional experiments utilizing pharmacological interventions.

Based on these studies, it became possible to model arrhythmogenic wave dynamics in human atrial tissue with high accuracy, including processes occurring during atrial fibrillation [39]. This was achieved by introducing a complex parameter responsible for tissue heterogeneity and a corresponding change in the wave propagation equation in the tissue. This parameter also determined the fractal dimension of the conducting structure. The paper investigated how the real and imaginary parts of the fractal dimension affect the character of the action potential and transmembrane ion currents, as well as the shape of the restitution curve. The formation of spiral waves and their shear trajectory were analyzed depending on the values entered. The created model makes it possible to simulate a wide range of wave phenomena in tissue that are correctly consistent with clinical practice and lays the foundation for a class of models of conduction in tissue, taking into account its heterogeneities in complex variables.

We also investigated the behavior of the wave at the excitability boundary [40]. In the models available in the literature, it is customary to set the Neumann conditions or current conditions to describe the effects at the culture boundary. However, the question of currents at the boundary of the excitability of the medium has not been studied. It is known that there are currents called demarcation or traumatic currents (or injury currents) directed to injured and no longer conductive tissue [41]. We considered the dependence of this current through the boundary. To obtain realistic boundary conditions, a series of experiments was carried out to characterize the magnitude of the flow through the boundary. Measurements were made of the propagation velocity of excitation waves in strips with a culture of cardiomyocytes with different widths in experiments on cell culture. Thus, an important result was obtained, in which the use of the Neumann boundary conditions is not always correct, and the consideration of leakage currents is necessary for the presence of traumatic defects, such as ischemic zones, infarct scars, or fibroblasts having gap junctions with cardiomyocytes.

These results significantly expanded the understanding of the mechanisms of formation and instability of spiral waves, which was used in the work [42]. In the work, based on the Fitzhugh–Nagumo model, the stability of conduction in tissue with heterogeneities under the occurrence of electromagnetic induction and even radiation was studied. The dependence of the stability of the conduction on the operation of various transmembrane currents and the effect of the influence of external factors on this conduction was investigated. The main result of the work is the obtained dependences of the stability of the conduction on the specified characteristics; in the future, they can be used for applied purposes for the termination of spiral waves.

Another paper [43] shows the influence of boundary conditions corresponding to the ablation performed on the atrial tissue on the occurrence and development of spiral reentry waves. The researchers found that as a result of the operation, the scar becomes a substrate for spiral waves and, on the contrary, generates arrhythmia in a new topology.

We also studied and modeled the boundaries, which are not lesions but areas with different excitability. Experimentally, we simulated the features of the wave propagation of a culture with cellular alignment and their boundaries. That is, areas of heart tissue where a different alignment of cells appears. We have shown in a previous work that cells are elongated along the fibers especially. It was shown that such a tissue has a different propagation velocity along and across the cells, and the authors note that taking this feature into account in the model will be of great importance for the engineering of heart tissues [44]. The study has significant value as it offers a method to control the anisotropy of both 2D and 3D structures. With the rapid development of tissue engineering, this study offers an accessible but efficient technique for creating highly organized and functional cardiac tissue. Subsequently, the architecture of the heart tissue was created in experimental models using this developed method.

An example of using the described technique can be the work of [45]. The main described result is a framework created based on polymer fibers for growing “patches” for cardiac tissue—small flat areas that can cover the non-conductive area and thus restore the conduction as a whole. When creating it, it is important to reconstruct the structure (anisotropy) of the tissue by the physiological one in the region of interest of the heart, which is possible with the use of such a framework even in the case of anisotropy in the third series, as described in the work. In addition, this class of polymer framework opens up the possibility for an impressive number of experimental works on the creation and study of three-dimensional in vitro models.

One such experimental model of excitation propagation across the border between regions with different cell orientations showed that when an excitation wave crossed the border between aligned regions, it changed its shape and acceleration [46]. The wave accelerated in the flattening direction before crossing the boundary and then accelerated in the horizontal direction after crossing. A more detailed analysis of the wave propagation in the boundary zone showed that it did not simply demonstrate a switch in propagation velocity. Depending on the direction of the transition between the zones, the wave approaching the boundary experienced either a delay or a jump in propagation. Before acceleration, the wave lingered at the boundary, and before deceleration, a propagation jump was observed, which is characteristic of the start of reentry. We thus showed that in the boundary zone, the wave velocity undergoes a nonmonotonic transition. We also performed statistical analysis to disprove the hypothesis that the occurrence of delays or hops is independent of propagation direction. The results showed an extremely unlikely case, supporting the hypothesis that the direction of propagation affects the occurrence of delays or jumps. We also showed the effect of refractory zones from previous waves on subsequent waves by observing unidirectional conduction blocks at the edge of the alignments.

This work was expanded by the following myocardium [47] study, which showed the development of wave dynamics in tissue with anisotropy, taking into account fibrosis. The main result of the work is the analysis of the influence of spatial heterogeneity of fibrosis, coupled with natural physiological anisotropy, on the probability of arrhythmia in the tissue. It was shown that this probability primarily depends on the maximum local level of fibrosis in the tissue; this is the main conclusion of the work. Also, in the work, a simulation of cardiac ablation in the tissue and the study of its effect on the wave dynamics in the tissue was carried out. Additionally, the influence of the size of heterogeneities on the parameters of arrhythmia in the tissue was analyzed.

One way to model the structural features of cardiac tissue is coculture. A striking example of such cell-based modeling is the work [48], where modeling of the fibrous region was carried out using human skeletal myotube and neonatal rat cardiomyocytes. These features can also be modeled in 3D, as shown in the work on the co-culture of cardiomyocytes with mesenchymal stem cells [49].

The structural features introduced into the generated biomimetic of cardiac tissue vary depending on the disease being modeled. Sometimes, the work uses cells that already have a congenital mutation, and the introduced defects or structural changes only enhance the effect. A summary with examples of work on the introduced structural features depending on the pathology being modeled is given in Table 1 and Table 2. We presented in Table 1 biomimetics that were created as structural pathologies but also added simulated congenital pathologies caused by mutations, such as long QT interval syndrome, hypertrophic cardiomyopathy, Duchenne muscular dystrophy, and others. These congenital pathologies illustrate that even when selecting a specific cell type, structural features play an important role, which is their modeling using substrate, co-culture, and other methods [50,51].

## 4. Application of Different Cell Types to Simulate Cardiac Tissue Models

Nowadays, we have different models of cells and cell cultures for studying cardiac tissue and excitation wave propagation. Let us consider some cardiomyocyte cultures that can be used to create a biomimetic of cardiac tissue.

First of all, this is a primary culture of animal cells, that is, a cell culture obtained by isolating cells of a certain type of tissue directly from an organ of a living organism (ex vivo) [95,96]. Cardiomyocytes cannot combine into excitable tissue after a certain period of their development. When cardiomyocytes are isolated in the postnatal period, the cells cease to form intercellular contacts and no longer form cardiac syncytium [97]. Therefore, it works with the compilation of a model of cardiac tissue; you will often find neonatal cardiomyocyte cells—these are cells obtained in the early period after the birth of the organism—when organs are still being formed; for example, in a rat, this period is no more than five days. Cells are isolated from the heart muscle using enzymes that degrade the extracellular matrix, such as trypsin. Primary cells have a limited lifespan outside the body. For neonatal rat cardiomyocytes, the optimal period for research will be 1–2 weeks.

Another possible solution for modeling cardiac tissue in an experiment is immortalized cell lines of cardiomyocytes [98]. We also used them in a number of our works. However, their main drawback is the degradation of cells with the number of passages [99,100]. The properties and functional features of cells change, which can greatly affect the results of experiments. In addition, as a rule, such cells have been obtained from tumors or modified to cause proliferation since, in adults, cardiomyocytes are highly resistant. That is, these cells could similarly be damaged even before the start of work with them.

In our works, the example of the use of an immortalized cell line illustrates well both the modeling of boundaries with different excitability and photocontrol methods [101]. Thus, we investigated the timing of heart cell cultures derived from different origins to study their electrical behavior and potential applications. We considered the effects arising at the border of cocultures of neonatal cells of different ages, as well as different animal origins. Using optical mapping, we studied the formation of a single syncytium in a coculture at the macro level, checking the transmission of excitation from cell to cell using fluorescent dyes.

The following cell material was used: cardiomyocytes of newborn rats and atrial mouse myocytes of the HL-1 line without transfection and transfected with the ChR-2 light-dependent ion channel. Cells were co-cultivated in polydimethylsiloxane (PDMS) masks, which were two round compartments connected by a thin channel, where the border between cell cultures was formed. Experimental results demonstrate successful synchronization of heart cell cultures of different origins. This synchronized model more closely mimics the behavior of the whole heart, showing different conductivities. It should be noted that cells of different origins were taken, taking into account their difference in excitability, which made it possible to create such a model.

That is, when building a model of cardiac tissue, we need to focus on real cases that we want to experimentally recreate. We must take into account the excitability, the currents of the cell channels, the animal, and so on. However, the question remains about the number of cells that are necessary for the successful conduction of the model of cardiac tissue. After all, both the speed of conduction and heterogeneity, the importance of which we emphasized, depend on the number of cells. So, we began to study the formation of the tissue maturity in vitro.

Answering the questions “How cardiomyocytes begin to form the heart tissue as a single conducting system after seeding, and how many minimally conductive cells are needed for its formation?”, we came to unexpected conclusions [102]. We solved the problem of percolation but for cardiac tissue. It is also important that in such a model with a minimum number of cells, we describe diffuse fibrosis in the heart and the mechanisms of arrhythmia, which are poorly understood. We took a comprehensive approach to this problem and combined experimental and computational research methods, taking into account the polygonal shape of cells.

To create a tissue model in this study, we used neonatal rat cardiomyocytes (NRVM). After adding non-conducting cells at planting, we generated the model itself, deriving cell number and conduction rate data from it for various numbers of non-conducting cells using optical mapping and immunocytochemical analysis. To describe the formation of a cell monolayer of cardiac tissue, we used the Potts cell model, which was described in detail in our previous work [103]. Within the framework of the model, two types of cells (conductive cardiomyocytes and nonconductive fibroblasts) acquire a morphology corresponding to experimental observations after a series of Monte Carlo steps, at each of which the cell shape can undergo energetically favorable changes. In this setting, the laws of interaction between the cell and the environment were adapted by the observed parameters of the cell. Additionally, in this study, adjustments were made to the formation of cell contacts between cardiomyocytes. As a result, the system reached an equilibrium state, which corresponded to experimental observations.

Thus, it was possible to achieve a very low percolation threshold observed in the experiment, which may indicate a significant positive effect of cell morphology on the nature of conduction in tissue and its parameters. We were able to show the paradoxical ability of tissue with an extremely high proportion of non-conductive cells (up to 75%) to conduct electrical signals and contract synchronously, while before this analysis, randomly distributed cells predicted a loss of connectivity by a maximum of 40%.

Adult cardiomyocytes are difficult to obtain and can be examined for a very short time, only in the form of sections of the heart or single cardiomyocytes. They, too, cannot re-arrange the conductive layer by an adult. Therefore, induced pluripotent stem cells or human embryonic stem cells are now used to obtain adult cardiomyocytes. They are unique in their ability to self-renew and differentiate into all types of tissues. The very state of pluripotency in vitro is maintained by a complex system of specific gene networks and signaling molecules [104]. The interaction of internal transcription factors, the central place among which is occupied by the Oct4, Sox2, and Nanog genes [105,106], and external signaling molecules form a characteristic state of the epigenome and a specific pattern of gene expression. It is the pattern of expression and epigenetics that begins to change during spontaneous or forced differentiation of stem cells. Scientists see great prospects in the use of iPSCs as they solve several problems inherent in embryonic stem cells, such as immune incompatibility in the case of transplantation and ethical difficulties.

**Figure 3:**
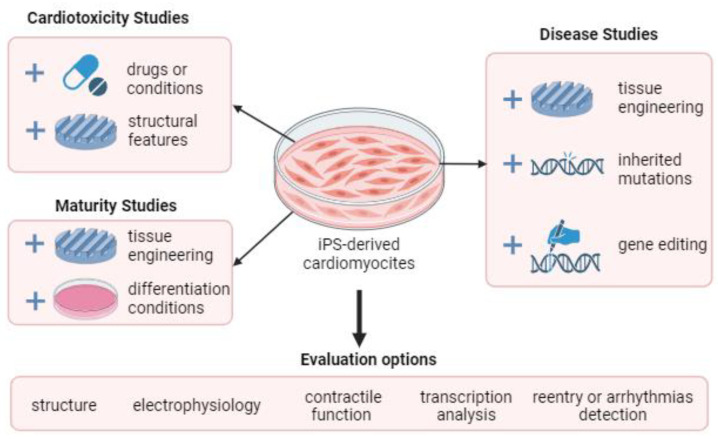
Diagram of the purposes for which a biomimetic of cardiac tissue is designed from iPS-derived cardiomyocytes.

Induced pluripotent stem cells can be used for many tasks in modern cardiology: cardiotoxicity, patient-specific study of pathologies, and the study of the processes of cell differentiation and tissue maturation. The main functions of cardiomyocytes induced from pluripotent stem cells are presented in Figure 3. Typically, the evaluation of any biomimetic of cardiac tissue, depending on the purpose of its creation, is based on the following parameters, which evaluate the proximity to real tissue: the resulting structure, electrophysiology, tissue maturity, etc.

Indeed, one of the most important obstacles in modeling heart disease or drug response using hPSC-CMs is their immaturity, both structural and functional [107]. Growth of the prenatal heart is primarily driven by the proliferation of cardiomyocyte progenitors, a process called hyperplasia. During postnatal development, cardiomyocytes exit the cell cycle and typically undergo a final round of DNA synthesis without cell division, generating large tetraploid nuclei [108,109].

Then, the cardiomyocytes grow rapidly, and the cell volume increases 10–20 times, which leads to hypertrophy of the muscle fibers. The profile of hPSC-CMs is known to be similar to that of prenatal cells in terms of structure, gene expression, energy, strength, conductance, ion channel density, and Ca2+ kinetics. Unlike mature human cardiomyocytes, immature cardiomyocytes do not have anisotropic, rod-shaped, ordered myofibrils, contractile cytoskeletons, T-tubules, DNA-rich nuclei, junctions, increased calcium signaling, and other organelles. Notably, components such as mitochondria can mature morphologically in proportion to the maturation of cardiomyocytes. Mitochondria in immature cardiomyocytes are round in shape, small in number and size, and located around the nucleus with a sparse density of cristae, whereas mitochondria in mature CMs are oval in shape, large in number and size, with a thick density of cristae. Moreover, such mature organelles are arranged in an orderly manner between myofibrils and under the sarcolemma [110].

There are models created specifically to study the maturity of cardiomyocytes. Usually, such models are 2D, but there are also 3D ones that demonstrate greater cell maturity. Thus, using tissue engineering modeling of cardioids created using non-adhesive substrates in a hydrogel, it was shown that WNT, ACTIVIN, and VEGF control the self-organization of CM in cardioids [72]. Sometimes, similar to modeling fibrosis or various parts of the heart with a complex structure, other cells are added to the model of cardiomyocyte maturity, designed to increase the maturation of cells in the process of differentiation. For example, it was found that cardiac fibroblasts promote structural maturation of hiPSC-CMs in generated microtissues [87].

Several groups of researchers came to the production of functional cardiomyocytes from iPSCs in vitro [111,112,113]. As mentioned earlier, during differentiation, a certain epigenome and transcriptome are established in cells, therefore, one of the most reliable and informative indicators of the completion of differentiation [114]. For example, in the early stages of differentiation, the expression profile of cells is very similar to that of neonatal cardiomyocytes. However, this has become an obstacle to the use of cardiomyocytes from iPSCs since the formation of a correct expression profile takes months [115,116]. To use human iPSCs and create heart tissue from them, we conducted several studies on the maturation of such cells into the adult phenotype of cardiomyocytes and, in general, the functional properties of differentiated cells.

One of our papers presents fundamental research on the electrical functionality of iPS cells that differentiate into cardiomyocytes [117]. We investigated the ability of cardiomyocytes derived from human iPSCs to form new syncytium on different days of differentiation, which was measured during the experiment using optical mapping and immunocytochemical analysis.

For this, differentiating cells were divided into two groups, namely the control group, which was not subjected to transfer during cultivation and formed naturally, and the transferred group, which was transferred to a new substrate on days of differentiation from the 12th to the 20th. We also studied the activity of potassium channels Ikr and Iks using E4031 as an Ikr blocker and isoprenaline as an Iks activator. The experiment showed a significant difference in the electrical properties of cells transplanted before and after day 20 in both lines. That is, the transfer of cells at an early stage of differentiation (before day 18) contributes to the almost complete restoration of syncytium, while in cells transferred on day 21, syncytium was heterogeneous.

The article revealed dynamic changes in the electrophysiological properties of differentiating cells, which occur in about a couple of days and fundamentally change the propagation of the wave in the tissue [117]. One important finding of the work is the identification of connexin 43 (Cx43) as a key protein responsible for electrical communication between differentiating cardiomyocytes, as it forms gap junctions. However, the paradox remains unresolved: Cx43 is more expressed at the late stages of differentiation, but, as the experiment in this article showed, the culture forms its electrophysiological properties at early stages. This paradox may lead to new theories for the formation of electrical bonds in differentiating cells, but it has not yet been elucidated. Studying the properties of cardiomyocytes differentiated from iPSCs also demonstrated that their use on the 50th day of differentiation is essentially relevant to the adult phenotype of cardiac tissue. Further, by combining the knowledge about heterogeneities, we obtained a full-fledged model for testing substances and studying the occurrence of reentry.

## 5. Arrhythmogenicity Test as an Application of Cardiac Tissue Biomimetic

Biomimetics of cardiac tissue can be used not only for research but also to detect potential cardiac side effects of drugs [118,119]. In the vast majority of modern experimental models that allow studying and analyzing cases of reentry on tissue from ventricular cardiomyocytes, wave breaks can occur on random inhomogeneities located throughout the sample [109,120]. Such a random spread of inhomogeneities was studied by us and described in the previous sections. The absolute randomness of wave breaks does not make it possible to analyze data on cases of violation of conduction in the sample, which is necessary to determine the mechanism for the occurrence of reentry since the data in such experiments are random and unsystematic, and the experiment itself is not reproducible.

To solve this problem, there are several standardized barriers or boundaries. We will focus on our experimental tissue engineering model with a standard inhomogeneity, which is a sharp cut in tissue with a standard thickness of about 30 µm, based on a human-induced pluripotent stem cell (iPSC)-derived cardiomyocyte layer [81]. The data from the developed model are based on a single systemic parameter, the measure of arrhythmogenicity. The measure of arrhythmogenicity introduced for our test is a probabilistic expression (number of occurrences) of any conduction disturbance at a given standard obstruction for different frequencies in cultured cells from different patients. One such value is calculated for each stimulation frequency on several samples from one patient.

It is more convenient to present measures of arrhythmogenicity in the form of a bar graph, in which, for different frequencies of electrode stimulation, three cases of excitation wave conduction on the model are recorded: (1) cases of normal conduction (without wave breaks); (2) the measure of arrhythmogenicity itself, as cases of reentry in the vicinity of a standard obstacle; and (3) cases of non-assimilation of the electrical stimulus by the tissue (the tissue does not respond to the applied impulses with the frequency of their supply). In all experiments, the wavefront propagated strictly perpendicular to the standard obstacle. In this study, we present a novel and promising approach to assess the arrhythmogenic potential of drug use. The study highlights the importance of developing more accurate and reliable methods for evaluating the proarrhythmic effects of drugs during preclinical drug development. We have shown that such results in such models correspond to real medical data on antiarrhythmics, which shows the possibility of their further use for testing [101]. Further investigations in this field could lead to improved drug testing protocols and contribute to enhanced patient safety.

On the example of the developed test for arrhythmogenicity, we showed the arrhythmogenic potential of cyclophosphamide, a commonly used chemotherapeutic agent, using human-induced pluripotent stem cell-derived cardiomyocytes (hiPSC-CMs) [102]. Cyclophosphamide is known to be associated with an increased risk of cardiac arrhythmias, but the underlying mechanisms and specific effects on cardiomyocytes are still not fully understood. Therefore, we choose hiPSC-CMs as an in vitro model to evaluate the effects of cyclophosphamide on cardiac electrophysiology. The study demonstrated that cyclophosphamide treatment resulted in prolonged action potential duration and increased incidence of early afterdepolarizations, two important markers of potential arrhythmia development.

We discussed the possibilities of detecting reentry on cell monolayers, but sometimes, it is important to see what happens exactly in 3D. Of course, it is not possible to make a complete heart model based on cells at this stage. Scientists are faced with vascularization problems and other limitations that cause cells to simply lose their properties or die. We will talk about substrates that can potentially lead to 3D modeling of cardiac tissue in the next section. The superimposition of thin sheets of cells on top of each other can also be used to create a biomimetic of cardiac tissue as a model of reentry arrhythmia.

In an example of such a study, we again used cardiomyocytes derived from human pluripotent stem cells [10]. Previous models have had limitations, mainly due to their inability to accurately model the complex, multi-layered structure of the human heart. The methods used in this study include creating layers of heart cells derived from human pluripotent stem cells. These cell sheets were built using a layer-by-layer methodology, creating a three-dimensional tissue structure that closely resembles the layers and organization of native human heart tissue. In the resulting sheets, a recurrent arrhythmia was induced in these layers of heart cells by manipulating various electrical and chemical factors.

Thus, we successfully generated reciprocal arrhythmias in cardiac cell layers derived from human pluripotent stem cells, reproducing the pathological condition observed in patients suffering from reentry arrhythmias. Importantly, the electrophysiological properties of the induced arrhythmias were very similar to those observed in human patients. This proves the effectiveness of the model in accurately simulating complex arrhythmias.

The presented arrhythmogenicity test is not the only research test for cardiotoxicity. The most common and standard test today is the hERG channel test (encoded by KCNH2) [121,122], but despite the mandatory requirement of this test, drugs show alarming cardiotoxicity after entering the market. A systematic analysis indicated that primary cardiotoxicity accounted for 74% of post-market withdrawals, such as arrhythmia (35%), cardiac injury (cardiomyopathy) (38%), and CV events (22%). In addition, during the preclinical to marketing and post-approval stages, CV problems accounted for 22% of withdrawals [109]. To solve this problem, a Comprehensive in vitro Proarrhythmia Assay (CiPA) initiative was established [123].

An international study across multiple sites evaluated the effectiveness of multielectrode arrays (MEAs) and hiPSC-CMs in predicting arrhythmia risks [124]. The study found these methods to be more accurate than current guidelines. The Japanese National Institute of Health Sciences is also working on developing a more precise clinical proarrhythmia risk prediction method called Japan iPS Cardiac Safety Assessment JiCSA [125]. Both CiPA and JiCSA initiatives have demonstrated that hiPSC-CMs can reliably assess proarrhythmia risk.

Additionally, many reports show that hiPSC cm evaluations can be useful for assessing drug-induced toxicity and guiding drug development. Long-term toxicity is a significant clinical issue, as it can be unpredictable. Researchers have used hiPSC-CMs to examine the cardiotoxic effects of both existing and emerging medications, including CCBs, anthracycline doxorubicin, and small-molecule kinase inhibitors [126,127].

Cardiovascular side effects are often caused by CM dysfunction, leading to a recent focus on contractile function as a safety assessment criterion. Studies have shown that hiPSC-derived engineered heart tissues (EHTs) can detect cardiac side effects not discovered in clinical trials. Despite the cost and production limitations of EHTs, scaffold-free microtissues offer promising prospects for large-scale drug screenings, and future miniaturized EHT systems may combine the advantages of both methodologies [128].

To better understand how models are designed for various purposes, we compiled Table 2, where biomimetics of cardiac culture are divided into three main subgroups: organoids, microtissues, and engineered models of cardiac tissue. All models were constructed using specific substrates and specific cells for different cardiac tissue model purposes. Having followed the available biomimetics, one can notice some patterns; 3D modeling often does not provide the structural features of the tissue and is based on the formation of embryoid bodies. Two-dimensional modeling, on the contrary, focuses on structural features, and it is possible to model specific pathologies using it (Table 2).

## 6. Variety of Substrates for Biomimetic Cardiac Tissue Models

Substrates for cell culturing are of great importance for the reliability of the cell model used for in vitro arrhythmia studies. Recreation and control of the microenvironment resembling in vivo heart conditions for two-dimensional in vitro models is not a trivial task, considering extracellular matrix (ECM) mechanical properties and spatial organization [129].

Typically, cardiac cells in 2D models are cultured on elastic hydrogels such as polyacrylamide (PAA) [104,105,106,107,108,109] and polydimethylsiloxane (PDMS) [128,129,130] gels with protein coatings. The use of such substrates helps to mimic the mechanical properties of cardiac ECM [131]. It has been shown that substrate stiffness of physiological (10–15 kPa) and fibrotic (30–100 kPa) values may stimulate [132] or inhibit the contractile function of cultured hiPSC-derived cardiomyocytes correspondingly [129,130,133].

As the cardiac tissue is principally anisotropic, isotropy or anisotropy of cell substrates plays a key role in the modeling of normal or pathological cardiac tissue. Anisotropic organization of substrates enforces alignment and elongation of cultured cardiomyocytes and leads to an increase in contraction force in both primary rat [134] and hiPSC-derived [106] cardiomyocytes. It was demonstrated substrate anisotropy also affects cell ultrastructure, resulting in more aligned myofibrils, Z-lines [130,134], and actin [131].

There are several approaches to introduce anisotropy organization on the substrate surface:

(1) microcontact patterning with proteins (i.e., fibronectin [132,134,135,136], laminin [136], collagen [137], Matrigel [130], and gelatin [131]);

(2) fabrication of contact guidance cues such as microgrooves 62 in the substrate;

(3) deposition of oriented nanofibers on the substrate surface.

We will discuss the latter method more thoroughly. Covering the initial substrate (i.e., PDMS) by oriented micro and nanofibers based on biocompatible polymers is one of the most prominent ways to organize anisotropic substrates for cardiac cells. It may be achieved by the use of the electrospinning technique [44], which allows for the efficient obtaining of fibers from a wide spectrum of polymer materials with various mechanical and biological properties with fiber diameters ranging from hundreds of nanometers to several microns [138,139,140,141,142,143,144,145,146,147].

This method may be effectively used to fabricate tissue-engineered constructs with cellular alignment and tissue architecture controlled on a micro-scale by adjustment of orientation and positioning density of electrospun nanofibers. Thus, the formation of continuous anisotropic cardiac tissue was demonstrated only on substrates with aligned nanofibers. Obtained tissue-engineered constructs behaved as unified excitable networks supporting electrical excitation wave propagation, resulting in the synchronization of contractile activity of cultured cardiomyocytes [44].

A wide range of biocompatible polymers may be used to create electrospun nanofiber substrates, such as polymethylglutarimide (PMGI) [44], poly(**ϵ**-caprolactone) (PCL) [148], poly(lactide-co-glycolide) (PLGA) [148], polyurethane [149], poly-L-lactic acid (PLLA) [149,150], and natural silk fibroin and recombinant spidroins, including ones containing and not containing RGDS motif [151] and polyvinyl alcohol/silk fibroin composite [152].

Remarkably, silkworm and spider silk proteins may be considered a very promising class of tissue engineering materials due to a combination of biocompatibility, exceptional mechanical properties, and suitability for electrospinning. It was demonstrated that scaffolds based on silk proteins may effectively facilitate the healing of skin wounds [153,154] and bone defects [155] in vivo and utilized for neural [156] and cardiac [157] tissue engineering in vitro. Analysis of excitation propagation in the cultured tissue shows the ability of the cardiomyocytes cultured on silk protein nanofiber substrates to form a functional cardiac syncytium [151]. Therefore, it may be concluded that the recombinant analogs of spider proteins, spidroins, are appropriate substrate materials for cardiac tissue engineering.

However, it must be noted that most of the abovementioned polymer materials require fibronectin coating to provide necessary cell adhesion properties. Here, we may benefit from the genetic modification availability of recombinant spidroins, as modified recombinant spidroins containing RGDS (rS2/12-Linker-RGDS) motif exhibit a level of cardiomyocyte adhesion comparable to the PCL and silkworm silk fibroin fibers coated by fibronectin [151].

One of the interesting applications of electrospun biopolymer fiber substrates is the possibility of culturing cardiomyocytes on suspended nanofibers. It was demonstrated that cardiomyocytes tend to create a ‘‘sheath” structure, enveloping suspended fiber [150]. Therefore, the cell–fiber contact area becomes much larger than the standard focal attachment model may predict. Moreover, a fragment of a single biocompatible polymer fiber of subcellular size may be used as a minimized “one-dimensional” polymer substrate itself. It was shown that fragments of PLLA or silk fibroin nanofibers with fibronectin coating may be used as effective scaffolds for solitary cardiomyocytes during cell transfer in vitro [158] and in vivo [159], supporting the organization of myofibrils and cytoskeleton of cells in suspension and restoring the cell excitability before adhesion to the recipient cell monolayer layer or tissue, respectively. Thus, such kernel substrates may be effectively used for in vitro modeling of cell transplantation processes in arrhythmia studies.

Cell transfer experiments in vivo show that the use of polymer kernel substrates leads to rapid electromechanical graft–host coupling: the synchronization of transplanted cells with isolated rat heart contractions (and consequently with each other) takes only 30 min after the first contact, while in the absence of any substrate, such a process may be chaotic, accompanied by engraftment arrhythmia, and would require several hours or even days [159].

One of the important aspects of the development of cardiac tissue models on various substrates is an analysis of substrate–cell interaction on a microscopic level. While conventional methods of optical or scanning electron microscopy mostly provide a view from above in the scaffold plane, techniques of nanoscale 3D reconstruction, such as “slice-and-view” scanning electron [160] or probe nanotomography [161,162], may provide more insights into cell–scaffold contact details. Both of these methods comprise sectioning (removing ultrathin layers on epoxy-embedded samples with the diamond knife of ultramicrotome) and examining the freshly sectioned surface with an electron beam or probe microscopy cantilever tip. Acquiring a series of consequent images after each section enables the reconstruction of 3D structures from layer-by-layer data. When the sectioning plane is perpendicular to the nanofiber substrate plane, the scanning probe nanotomography approach may be effectively used for the 3D reconstruction of nanofiber scaffolds [153,154] and the determination of volume porosity, surface-area-to-volume ratio, and other 3D morphological parameters. Reconstructions of the 3D structure of cell–substrate constructs enable the assessment of topology and calculation of quantitative and morphological characteristics of cells and cell–fiber interfaces, which are important in determining the cell condition and biological activity [150,163].

**Figure 4:**
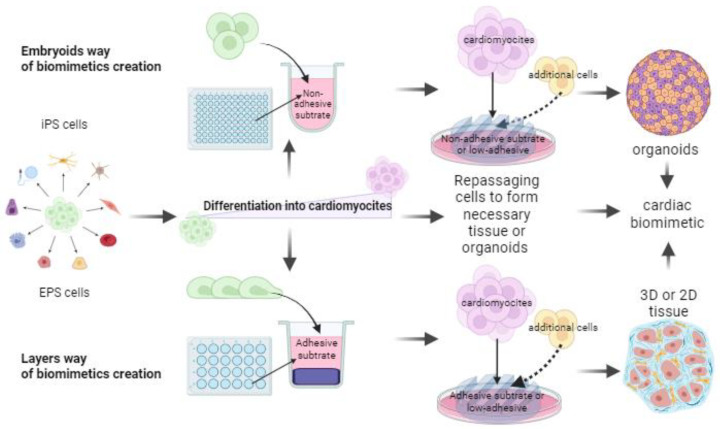
Different ways of constructing biomimetic cardiac tissue by differentiation of stem cells on different substrates.

Essentially, using substrates and masks made of polymers, most often PDMS, with varying degrees of adhesion, you can create very different 3D structures from tissues to organoids and chambers [71]. The general concept of constructing such structures is presented in Figure 4. The only thing that is not shown in the figure is the use of hydrogels. With the help of them and other proteins, it is possible to create even highly organized organelles, as in the work [80], where scientists have created a universal platform that allows the creation of electrophysiologically diverse tissues of the atria and ventricles, capable of providing multi-month biophysical stimulation of three-dimensional tissues for polygenic disease modeling. This platform allows the growth of thin cylindrical tissues suspended between two parallel poly(octamethylene maleate-(anhydride) citrate) (POMaC) polymer wires. During the process, they also used PDMS masks to form micropatterns. To obtain the cells, a directed differentiation protocol and electrical conditioning were used, and the specification of the atria and ventricles can be reliably achieved.

There are important works on creating structured organoids or disease models in 3D, combining the two methods described in Figure 4 [66]. For example, in one of these studies, adhesive substrates were used, which were gradually applied along with cells onto an embryoid body that differentiated into cardiomyocytes [73]. More and more new cells were applied to the substrates, and substrates were again applied on top, which led to the creation of an embryoid where all the cells were found in certain layers.

In some studies, the effect of the folds and twists of the substrate on the conduction in the fabric was shown [164]. It was discovered that spontaneous contractions in such a monolayer were more frequent than in a flat one, which gives such systems a high potential in the design of bioreactors.

A good illustration of the high potential of microfiber structures for regenerative medicine is the following work [67]. In it, the researchers managed to create a rat ventricle frame and populate it with heart cells. Such a structure created small reductions, initiating fluid emissions. The structure has been investigated and described as a promising model for regenerative therapy in general.

Another important direction in creating a cardiac biomimetic is the use of extracellular matrix (ECM) hydrogel. This matrix makes it possible to create structural organoids for studying cardiac pathologies and partially solves the problems of vascularization [52,65]. By the way, it is precisely this problem that often does not lead to successful attempts to create 3D biomimetics. However, some groups solve it in rather elegant ways, for example, by stretching groups of cells differentiated in embryonic bodies [68].

## 7. Discussion

The presented review covers the main aspects of the generation of cardiac tissue biomimetics. A biomimetic of cardiac tissue as a model can serve different research goals: the influence of external factors on cardiac tissue, the role of heterogeneities in cardiac tissue, and the features of congenital or acquired pathologies. For different purposes, it is necessary to choose different ways of not only choosing materials but also structural features. For example, for testing substances, the review presented a tissue engineering research model of human heart arrhythmias. We also showed in the review that sometimes, it is necessary to involve computer modeling to study the main factors in the development of cardiac arrhythmias, for example, for calculating the minimum number of cells required for conduction or as a reinforcement for testing substances for cardiotoxicity.

One to several types of cell cultures can be used as a material for creating models: neonatal rat cardiomyocytes, immortalized HL-1 culture, including those with transfected channel rhodopsin, patient-specific human iPSC lines, and others. Cardiac arrhythmia is any deviation of the heart rate from normal sinus rhythm. The main cause of arrhythmias in the heart muscle is the appearance of a unilateral block followed by the formation of a spiral wave or reentry, which is a wave rotating in a circle with a frequency independent of the main pacemaker. Considering one of the causes of reentry, we can create the biomimetics of cardiac tissue necessary for the study of a specific cause, simulating these processes on them. We placed a special emphasis on heterogeneities and their role in the occurrence of reentry. The decisive role of inhomogeneities was noted after modeling the so-called free reentries and regulation of excitability in the resulting models.

In studies, it was found that the most likely mechanism for the occurrence of reentry in the heart tissue is the interaction of propagating excitation waves with inhomogeneity when the critical frequency of the waves is exceeded. It was known that a sharp increase in heart rate can lead to episodes of tachyarrhythmia, often turning into fibrillation. It has been hypothesized that the formation of reentry in such a system may occur due to inhomogeneities in the refractoriness of the cardiac tissue [165] or due to stimulation in the so-called “vulnerable window” [166]. Simulation of the process of rhythm change on the layers of cultured cardiomyocytes showed the failure of the “vulnerable window” hypothesis since, without external stimulation, this effect cannot be achieved. Thus, for a comparative analysis of the arrhythmogenic effect of various drugs, as well as a comparison of the probability of reentry formation in layers of healthy cells and heart cells with channelopathy, it is necessary to use models that describe the interaction of excitation waves with standardized heterogeneity. Such a model was proposed as an example of a tissue engineering solution for the study of arrhythmias. The model is a layer of cardiomyocytes differentiated from iPSCs and self-organizing into a kind of heart tissue (cardiac syncytium) with standardized parameters.

In the vast majority of modern experimental models that allow studying and analyzing cases of reentry on tissue from ventricular cardiomyocytes, wave breaks can occur on random inhomogeneities located throughout the sample. This complicates the interpretation of data on cases of violation of conduction in the sample, which is necessary for a clear definition of the wave-breaking mechanism. Therefore, new experimental tissue-engineering models are necessary. Our describing test was supplemented with a standard inhomogeneity, which is a sharp cut in tissue with a standard thickness of about 30 μm. The important role of the standard inhomogeneity lies in the fact that it was in its vicinity that cases of reentry occurrence were recorded and taken into account both in healthy tissue and in tissue with induced inhomogeneity. The success of the described test shows how important it is to take into account all factors when preparing a cardiac tissue biomimetic, especially structural features.

## Figures and Tables

**Table 1:** Classification of the possibility of creating cardiac tissue biomimetics depending on modeling tasks, namely, depending on the chosen pathology, using examples.

Type of Arrhythmias or Disease	Dimension	Basic Methods of Creating Heart Biomimetic	Cell Type and Gene Mutations	Substrate	Structural Features	Article
Ventricular tachycardias and Postmyocardial infarction	2D	Coculturing, non-adhesive, and adhesive micropatterning	Neonatal rat ventricular myocytes	fibronectin	Model of the infarct border zone with human skeletal myotubes, nonuniform anisotropic architecture	Chang, Marvin G. et al. “Spiral waves and reentry dynamics in an in vitro model of the healed infarct border zone.” Circulation research 105.11 (2009): 1062–1071. [48]
	3D	Coculturing	hiPSCs (836B3 line18), mesenchymal cell	Gelatine; 48-multiwell UpCell	3D cardiac tissue sheets with two cell types	Kawatou, Masahide et al. “Modelling Torsade de Pointes arrhythmias in vitro in 3D human iPS cell-engineered heart tissue.” Nature communications 8.1 (2017): 1078. [49]
	3D	Molds with ECM pre-gel	hiPSC (from patient with LQTS 2 and CPVT2)	Matrigel	3D cardiac rings on passive-stretcher devices	Goldfracht, Idit et al. “Engineered heart tissue models from hiPSC-derived cardiomyocytes and cardiac ECM for disease modeling and drug testing applications.” Acta biomaterialia 92 (2019): 145–159. [52]
	2D	Glucose-free and hypoxic conditions	Neonatal rat myocytes	Gelatine	Model of Ischemia/Reperfusion Injury, isotropic architecture	Sugiyama, A.; Shimizu, Y.; Okada, M.; Otani, K.; Yamawaki, H. Preventive Effect of Canstatin against Ventricular Arrhythmia Induced by Ischemia/Reperfusion Injury: A Pilot Study. Int. J. Mol. Sci. 2021, 22, 1004. https://doi.org/10.3390/ijms22031004 [53]
	3D	Polyacrylamide hydrogels with variable stiffness	iPSC: line TaP4	Gelatine	Engineered heart tissues (EHTs); different matrixes	Heras-Bautista, Carlos O. et al. “Cardiomyocytes facing fibrotic conditions re-express extracellular matrix transcripts.” Acta biomaterialia 89 (2019): 180–192. [54]
	2D	Coculturing (hiPSC-aCMs and hiPSC-vCMs)	hiPSCs: WiCell and IMR90-1	Matrigel	Non-structured monolayers	Gunawan, Marvin G. et al. “Drug screening platform using human induced pluripotent stem cell-derived atrial cardiomyocytes and optical mapping.” Stem Cells Translational Medicine 10.1 (2021): 68–82. [55]
Brugada syndrome	2D	Brugada syndrome associated line investigation	iPSCs lines (BrS1-iPSCs, Ctrl1-iPSCs, Ctrl2-iPSCs, and BrS2-iPSCs)	Geltrex	Non-structured monolayers	Li Wener, W., Stauske, M., Luo, X., Wagner, S., Vollrath, M., Mehnert, C.S. et al. (2020). Disease Phenotypes and Mechanisms of iPSC-Derived Cardiomyocytes from Brugada Syndrome Patients with a Loss-Of-Function SCN5A Mutation. Front. Cell Dev. Biol. 8, 592893. Epub 2020/11/17. doi:10.3389/fcell.2020.592893 [51]
		Brugada syndrome associated line investigation	hiPSCs: he BrS cell lines isBrSd1.9 (GOEi098-A.9), isBrSd1.12 (GOEi098-A.12), and isBrSd1.23 (GOEi098-A.23)	Matrigel	Non-structured monolayers	El-Battrawy, Ibrahim et al. “A cellular model of Brugada syndrome with SCN10A variants using human-induced pluripotent stem cell-derived cardiomyocytes.” EP Europace 21.9 (2019): 1410–1421. [56]
Short QT interval syndrome	2D	SQTS1 associated line investigation	hiPSCs: with N588K mutation in the KCNH2 gene and healthy hiPSC-CMs	Matrigel	Non-structured monolayers	Shinnawi, Rami et al. “Modeling reentry in the short QT syndrome with human-induced pluripotent stem cell–derived cardiac cell sheets.” Journal of the American College of Cardiology 73.18 (2019): 2310–2324. [57]
	2D	Embryoid body (EB)-based differentiating technology; SQTS1 associated line investigation	hiPSCs: SQTS (N588K KCNH2 mutation), isogenic control, and healthy control	Non-adhesive v-shaped plate; Matrigel	Atrial cell sheets (hiPSC-ACSs), non-structured monolayers	Shiti, Assad et al. “Utilizing human induced pluripotent stem cells to study atrial arrhythmias in the short QT syndrome.” Journal of Molecular and Cellular Cardiology 183 (2023): 42–53. [58]
	2D	SQTS1 associated line investigation	hiPSCs: with mutation (N588K) in KCNH2 and two healthy controls	Matrigel	Cell sheets (hiPSC-ACSs), non-structured monolayers	El-Battrawy, I., Lan, H., Cyganek, L., Zhao, Z., Li, X., Buljubasic, F. et al. (2018). Modeling Short QT Syndrome Using Human-Induced Pluripotent Stem Cell-Derived Cardiomyocytes. J. Am. Heart Assoc. 7, 7. Epub 2018/03/27. doi:10.1161/JAHA.117.007394 [59]
Long QT interval syndrome	3D	Decellularization for matrix creation	hiPSCs: line with LQTS2 and line with polymorphic ventricular tachycardia type 2 (CPVT2)	ECM pre-gel solution	ECM-derived engineered heart tissues (ECM-EHTs); anisotropic architecture	Giacomelli E. et al. “Cardiac microtissues from human pluripotent stem cells recapitulate the phenotype of long-QT syndrome.” Biochemical and Biophysical Research Communications 572 (2021): 118–124. [60]
	3D	Coculturing (three cell types); embryoid body (EB)-based culturing	hiPSC: wild-type (WT) hiPSC line LUMC0020iCTRL-06 (female, LUMCi028-A), and long-QT syndrome type 1 (LQT1) hiPSC line LUMC0021iKCNQ-30 (female, LUMCi039-A) carrying the KCNQ1-R594Q	Non-adhesive v-shaped plates; Matrigel-coated multielectrode array (MEA)	Cardiac microtissues	Goldfracht, I., Efraim, Y., Shinnawi, R., Kovalev, E., Huber, I., Gepstein, A., ... & Gepstein, L. (2019). Engineered heart tissue models from hiPSC-derived cardiomyocytes and cardiac ECM for disease modeling and drug testing applications. Acta biomaterialia, 92, 145–159. [52]
Hypertrophic cardiomyopathy (HCM)	2D	HCM-associated line investigation	hiPSC: wild-type (MYH7WT/WT), heterozygous (MYH7WT/MUT) and homozygous (MYH7MUT/MUT)	Matrigel	Monolayers	Bhagwan, Jamie R. et al. “Isogenic models of hypertrophic cardiomyopathy unveil differential phenotypes and mechanism-driven therapeutics.” Journal of molecular and cellular cardiology 145 (2020): 43–53. [61]
	2D	HCM-associated line investigation	hiPSC: TNNI3R21C/+, TNNT2R92Q/+ and MYH7R403Q/+	Matrigel	Monolayers	Margara, Francesca et al. “Mechanism based therapies enable personalised treatment of hypertrophic cardiomyopathy.” Scientific Reports 12.1 (2022): 22501. [62]
Duchenne muscular dystrophy (DMD)	3D	Embryoid body-based differentiation	iPSC: with DMD and control line	Non-adhesive plate	Non-structured organoids	Lin, Bo et al. “Modeling and study of the mechanism of dilated cardiomyopathy using induced pluripotent stem cells derived from individuals with Duchenne muscular dystrophy.” Disease models & mechanisms 8.5 (2015): 457–466. [51]
Arrhythmogenic cardiomyopathy (ACM)	2D	ACM-associated line investigation; coculturing	hiPSC: human PKP2 c.2013delC, PKP2 c.1849C>T, and control lines	Geltrex	Monolayers, spontaneous fibro-adipose cell differentiation	Kohela, A.v.K.S., Moens, T., Wehrens, M., Molenaar, B., Boogerd, C.J., Monshouwer-Kloots, J. et al. (2021). Epicardial Differentiation Drives Fibro-Fatty Remodeling in Arrhythmogenic Cardiomyopathy. Sci. Transl Med. 2021, 13. doi:10.1126/scitranslmed.abf2750 [63]
	3D	Generating EHT by PDMS; monolayer-based differentiation; and ACM-associated line investigation	ESC; hiPSC	PDMS strips; Pluronic F-127; collagen I; Matrigel	Micropatterning; engineered heart tissue (EHT)	Bliley, Jacqueline M. et al. “Dynamic loading of human engineered heart tissue enhances contractile function and drives a desmosome-linked disease phenotype.” Science translational medicine 13.603 (2021): eabd1817. [64]

**Table 2:** Classification of biomimetics of cardiac tissue and options for their design by examples.

Basic Heart Tissue Model	Purpose of Cardiac Tissue Modeling	Dimension	Cell Types or Cell Source	Substrate Types	Modeling of Structural Features	Article Examples
Organoids and organs-on-chip	Disease modeling and drug testing applications	3D	hiPSC lines and LQTS2 and CPVT hiPSC lines for ventricular-like cardiomyocytes	Decellularized porcine cardiac tissues (ECM); solubilized porcine cardiac ECM (pcECM) hydrogel	Non-structured ECM hydrogel	Goldfracht, I., Efraim, Y., Shinnawi, R., Kovalev, E., Huber, I., Gepstein, A., ... & Gepstein, L. (2019). Engineered heart tissue models from hiPSC-derived cardiomyocytes and cardiac ECM for disease modeling and drug testing applications. Acta biomaterialia, 92, 145–159. [52]
		3D	hESC line for ventricular and atrial cardiomyocytes	Decellularized porcine cardiac tissues (ECM); solubilized porcine cardiac ECM (pcECM) hydrogel	Non-structured ECM hydrogel	Goldfracht, Idit et al. “Generating ring-shaped engineered heart tissues from ventricular and atrial human pluripotent stem cell-derived cardiomyocytes.” Nature communications 11.1 (2020): 75. [65]
		3D	hiPSC-CMs (iCell Cardiomyocytes, Cellular Dynamics)	Non-adhesive agarose hydrogel molds	Non-structured organoids (50% hiPSC-CMs and 50% non-myocyte)	Richards D.J. et al. “Human cardiac organoids for the modelling of myocardial infarction and drug cardiotoxicity.” Nature Biomedical Engineering 4.4 (2020): 446–462. [66]
		3D	hiPSC-CMs and NRVMs	Polycaprolactone (PCL)/gelatin nanofibers	Structured ventricle chamber, anisotropic myocardial tissue	MacQueen, Luke A. et al. “Addendum: A tissue-engineered scale model of the heart ventricle.” Nature Biomedical Engineering 6.11 (2022): 1318–1318. [67]
	The vascularization in vitro problem	3D	hiPSC-derived cardiomyocytes; rat primary cardiac microvascular endothelial cells (CECs)	Organoid culturing in microwells mixed in Matrigel	Stress-structured vascularization	Ghosheh, Mohammad et al. “Electro-metabolic coupling in multi-chambered vascularized human cardiac organoids.” Nature biomedical engineering (2023): 1–21. [68]
		3D	hiPSC: hiPSCs from human foreskin fibroblasts	Ultra-low attachment Petri dish; 10% Matrigel in medium	Non-structured heart organoids, vascularized in vivo (after injection)	Lee, Seul-Gi et al. “Generation of human iPSCs derived heart organoids structurally and functionally similar to heart.” Biomaterials 290 (2022): 121860. [69]
		3D	hiPSC: H9 and H13 cell lines	Matrigel; U-bottom culture plate	Non-structured embryoids with vascularisation	Liang, Po-Yu et al. “Wnt signaling directs human pluripotent stem cells into vascularized cardiac organoids with chamber-like structures.” Frontiers in Bioengineering and Biotechnology 10 (2022): 1059243. [70]
	Investigation of cell maturation in vitro	3D	hiPSC: WTC hiPSCs (from B.R. Conklin lab, Gladstone Institute of Cardiovascular Disease; also available from the Coriell Institute NIGMS Human Genetic Cell Repository, cat. no. GM25256)	Micropatterned PEG substrate: Photomasks; SU8 patterns on silicon wafers; PDMS; PEG nonfouling thin polymer film; Covering: hESC-qualified Matrigel	Well-structured micropatterns: small patterned organoids in different forms (circles, triangles, and squares)	Hoang, Plansky et al. “Generation of spatial-patterned early developing cardiac organoids using human pluripotent stem cells.” Nature protocols 13.4 (2018): 723–737. [71]
		3D	Human cardiac microtissues (3D InSightTM Human Cardiac Microtissues from InSphero) and human biopsies	AggreWell400 plates, ultra-low attachment 96-well plates, hydrogel	Non-structured cocultured cardioids	Hofbauer, Pablo et al. “Cardioids reveal self-organizing principles of human cardiogenesis.” Cell 184.12 (2021): 3299–3317. [72]
		3D	hESC: HES3 NKX2.5–eGFP, HSC_ADCF_SeV-iPS, HES3 MIXL1–GFP, and HES3 NKX2.5–eGFP/eGFP (NKX2.5-KO) cell lines	U-shaped ultra-low attachment 96-well plate, Matrigel droplet per each well, and Geltrex (growth factor-reduced Matrigel preparation) or collagen I for embedding	High-structured heart-forming organoids (HFO) with three layers: IC (inner core with mesendoderm and cardiac-like cells), ML (NKX2.5–eGFP-positive myocardial layer), and OL (outer layer with mesendoderm)	Drakhlis, Lika et al. “Human heart-forming organoids recapitulate early heart and foregut development.” Nature Biotechnology 39.6 (2021): 737–746. [73]
		3D	ESC: atrial tissue (chicken embryos)	Polyacrylamide (PA) gels	Substrate micropatterning, Microstructured hydrogels	Thomas, Kandace et al. “Adherens junction engagement regulates functional patterning of the cardiac pacemaker cell lineage.” Developmental cell 56.10 (2021): 1498–1511. [74]
	Investigation of mechanotransduction-related events in vitro	3D	MSC: immortalized adipose-derived mesenchymal stem cells	Poly-L-lysine-g-poly and light-responsive substrates: Poly-Disperse Red 1-methacrylate (pDR1m)	Substrate micropatterning	Cimmino, C., Netti, P.A., & Ventre, M. (2022). A switchable light-responsive azopolymer conjugating protein micropatterns with topography for mechanobiological studies. Frontiers in Bioengineering and Biotechnology, 10, 933410. [75]
		2D, 3D	hESC: line CCTL14	Agarose gel	Non-structured embryoid bodies (“beating clusters”)	Caluori, Guido et al. “Simultaneous study of mechanobiology and calcium dynamics on hESC-derived cardiomyocytes clusters.” Journal of Molecular Recognition 32.2 (2019): e2760. [76]
		3D	hESC: line CCTL14 and hiPSC line reprogrammed from fibroblasts of a patient affected by Duchenne muscular dystrophy (DMD)	Laminin and fibronectin	Non-structured embryoid bodies (“beating clusters”)	Caluori, Guido et al. “Non-invasive electromechanical cell-based biosensors for improved investigation of 3D cardiac models.” Biosensors and Bioelectronics 124 (2019): 129–135. [77]
Engineered heart tissue (EHT)	Disease modeling and drug testing applications	2D, 3D	hiPSC: Ctrl cell lines (C25, ERC18, and ERC1)	Geltrex for monolayer cultures, fibrinogen and thrombin for EHT	Non-structured isotropic EHT between elastic silicone posts for contractile detection	Lemme, Marta et al. “Atrial-like engineered heart tissue: an in vitro model of the human atrium.” Stem cell reports 11.6 (2018): 1378–1390. [78]
		2D	hESC: human embryonic cell line HES2 (ESI, NIH code ES02)	Polydimethylsiloxane (PDMS) mold, microgrooving by photolithography	Substrate micropatterning, anisotropic sheet	Shum, Angie MY et al. “A micropatterned human pluripotent stem cell-based ventricular cardiac anisotropic sheet for visualizing drug-induced arrhythmogenicity.” Advanced Materials 29.1 (2017): 1602448. [79]
		3D	hESC: lines HES2 (female) and HES3-NKX2-5eGFP/w (female); hiPSC: hiPSC line BJ1D (male), C2A hiPSC derived cardiomyocytes, and iCell and iCell2 cardiomyocytes; patient biopsies	Collagen hydrogel, negative polydimethylsiloxane (PDMS) master mold, and PDMS master for microwell creation	High-structured micropatterning cell sheet with two ends: atrial and ventricle with poly(octamethylene maleate-(anhydride) citrate) (POMaC) polymer wires on each end	Zhao, Yimu et al. “A platform for generation of chamber-specific cardiac tissues and disease modeling.” Cell 176.4 (2019): 913–927. [80]
		2D	iPSC: iSMA6L cell line	Geltrex	Isotropic cell sheets with standard sharp obstacle	Slotvitsky, M., Tsvelaya, V., Frolova, S., Dementyeva, E., & Agladze, K. (2019). Arrhythmogenicity test based on a hu-man-induced pluripotent stem cell (iPSC)-derived cardiomyocyte layer. Toxicological Sciences, 168(1), 70–77. [81]
	Investigation of cell maturation in vitro	3D	hiPSC: line DiPS 1016SevA (from skin fibroblasts) and line Huv-iPS4F1 (from human umbilical cord vein endothelial cells (HUVECs))	Hydrogel GelMA and PEG-RGD	Non-structured monolayers	Acun, Aylin, Trung Dung Nguyen, and Pinar Zorlutuna. “In vitro aged, hiPSC-origin engineered heart tissue models with age-dependent functional deterioration to study myocardial infarction.” Acta biomaterialia 94 (2019): 372–391. [82]
		2D	NRVM cells	Polydimethysiloxane (PDMS, Dow Corning) tissue molds; PTFE template; fibrinogen, Matrigel	Structured cell/hydrogel tissue patches	Jackman, Christopher P. et al. “Engineered cardiac tissue patch maintains structural and electrical properties after epicardial implantation.” Biomaterials 159 (2018): 48–58. [83]
		3D	hESC: H9, WiCell lines; hESC-EPIs and hESC-derived CMs	Polydimethylsiloxane (PDMS); Collagen gel with Collagen I Rat Protein, Geltrex	Non-structured cocultural ETH	Bargehr, Johannes et al. “Epicardial cells derived from human embryonic stem cells augment cardiomyocyte-driven heart regeneration.” Nature biotechnology 37.8 (2019): 895–906. [84]
Microtissues	Disease modeling and drug testing applications	3D	iPSC-derived cardiomyocytes, Cellular Dynamics International, fetal human cardiac fibroblasts from Sigma (Cell Applications)	GravityTRAP plates	Microtissues with fibrosis modeling; cell cocultural aggregates (CM microtissues with fibroblasts)	Błyszczuk, Przemysław et al. “Activated cardiac fibroblasts control contraction of human fibrotic cardiac microtissues by a β-adrenoreceptor-dependent mechanism.” Cells 9.5 (2020): 1270. [85]
		3D	Neonatal mouse cardiomyocytes (NMVCMs)	PDMS spacers with methacrylated gelatin (GelMA); hyaluronic acid glycidyl methacrylate (HAGM) Photoinitiator lithium phenyl-2,4,6-trimethylbenzoylphosphinate (LAP) and PEGDA between	Substrate micropatterning: 3D methacrylated gelatin (GelMA) scaffolds patterned via Microscale Continuous Optical Printing (μCOP)	Liu, Justin et al. “Direct 3D bioprinting of cardiac micro-tissues mimicking native myocardium.” Biomaterials 256 (2020): 120204. [86]
		3D	hiPSC-CMs, hiPSC-cECs, and hiPSC-cFswild-type (WT) hiPSC line LUMC0020iCTRL-06, long-QT syndrome type 1 (LQT1) hiPSC line LUMC0021iKCNQ-30	Non-adhesive v-shaped plates; Matrigel-coated multielectrode array (MEA)	Non-structured microtissues	Giacomelli, Elisa et al. “Cardiac microtissues from human pluripotent stem cells recapitulate the phenotype of long-QT syndrome.” Biochemical and Biophysical Research Communications. 572 (2021): 118–124. [60]
	Investigation of cell maturation in vitro	3D	hiPSC-CMs and hiPSC-cardiacECs, hiPSC-CFs (CMFs), and Dermal Fibroblasts	V-bottomed 96-well microplates (Greiner bio-one)	Non-structured spheroids, 5000 cells each (85% cardiomyocytes and 15%endothelial cells)	Giacomelli, Elisa et al. “Human-iPSC-derived cardiac stromal cells enhance maturation in 3D cardiac microtissues and reveal non-cardiomyocyte contributions to heart disease.” Cell stem cell 26.6 (2020): 862–879. [87]
		3D	hESCs (WiCell line)	6-well ultra-low attachment plates	Non-structured spheroids with fibrosis modeling; coculturing	Lee, Mi-Ok et al. “Modelling cardiac fibrosis using three-dimensional cardiac microtissues derived from human embryonic stem cells.” Journal of biological engineering 13.1 (2019): 1–17. [88]
		3D	iPSC: human iPSC cell line DF 19-9-7 T (iPS, karyotype: 46, XY)	12-well Matrigel-coated plates	hiPSC-derived 3D organotypic cardiac microtissue (hOCMT); long-term culturing	Ergir, Ece et al. “Generation and maturation of human iPSC-derived 3D organotypic cardiac microtissues in long-term culture.” Scientific Reports 12.1 (2022): 17409. [89]
	Investigation of mechanotransduction-related events in vitro	3D	NIH 3T3 fibroblasts (American Type Culture Collection, ATCC)	PDMS with Pluronic F127 and collagen; fibronectin	Well-structured sheets; Substrate micropatterning	Legant, Wesley R., Christopher S. Chen, and Viola Vogel. “Force-induced fibronectin assembly and matrix remodeling in a 3D microtissue model of tissue morphogenesis.” Integrative Biology 4.10 (2012): 1164–1174. [90]
		3D	hCM: AC16 (a human cardiomyocyte cell line; Merck Millipore, Burlington, MA, USA) and hCF (a human normal cardiac fibroblast-ventricular cell line; Lonza, Basel, Switzerland) cells	Cell culture dish	Structured sheets by magnetic torque stimulation (MTS) system: hCM was seeded with magnet nanoparticles conjugated with WGA	Song, M.; Kim, J.; Shin, H.; Kim, Y.; Jang, H.; Park, Y.; Kim, S.-J. Development of Magnetic Torque Stimulation (MTS) Utilizing Rotating Uniform Magnetic Field for Mechanical Activation of Cardiac Cells. Nanomaterials 2020, 10, 1684. https://doi.org/10.3390/nano10091684 [91]
		3D	Human fetal cardiac fibroblasts (hfCF)	Spin-coated with SU8-2050 silicon wafers, PDMS	Step-profile steel fibrosis-on-chip platform	Gartner, Tom CL Bracco et al. “Cyclic strain has antifibrotic effects on the human cardiac fibroblast transcriptome in a human cardiac fibrosis-on-a-chip platform.” Journal of the Mechanical Behavior of Biomedical Materials 144 (2023): 105980. [92]
	Investigation of biomarkers	2D, 3D	hiPS-CMs (iCell™ Cardiomyocytes), primary human cardiac microvascular endothelial cells (hCMECs), and primary human cardiac fibroblasts (hCFs)	Ultra-low attachment spheroid microplates (Corning, 3830); CellCarrier-384 Ultra microplates	Non-structured microtissues; coculturing; and modeling fibrosis	Archer, Caroline R. et al. “Characterization and validation of a human 3D cardiac microtissue for the assessment of changes in cardiac pathology.” Scientific reports 8.1 (2018): 10160. [93]
		3D	iPS cells: human cardiac microtissues (3D InSightTM Human Cardiac Microtissues from InSphero) and human biopsies	Non-adhesive plates	Non-structured microtissues; coculturing; and modeling fibrosis	Nguyen, Nhan et al. “Translational proteomics analysis of anthracycline-induced cardiotoxicity from cardiac microtissues to human heart biopsies.” Frontiers in Genetics 12 (2021): 695625. [94]

## Data Availability

No new data were created or analyzed in this study. Data sharing is not applicable to this article.

## Abbreviations

ATP	Antitachycardia or overdrive pacing
CM	Cardiomyocytes
NRVM	Neonatal rat ventricular myocytes
iPSC	Induced pluripotent stem cells
hiPSC	Human induced pluripotent stem cells
iPS-CM	(iPSC)-derived cardiomyocytes
ESC	Embryonic stem cells
EHT	Engineered heart tissues
PMGI	Polymethylglutarimide
PCL	Poly(ϵ-caprolactone)
PLGA	Poly(lactide-co-glycolide)
PAA	Polyacrylamide
PDMS	Polydimethylsiloxane
PLLA	Poly-L-lactic acid
POMaC	Poly(octa methylene maleate-(anhydride) citrate)
ECM	Extracellular matrix
EPI cells	Epicardial cells
MEA	Multielectrode array
CPVT	Catecholaminergic polymorphic ventricular tachycardia
